# 1,3,5-Triazine: A Promising Molecular Scaffold for Novel Agents for the Treatment of Alzheimer’s Disease

**DOI:** 10.3390/ijms26030882

**Published:** 2025-01-21

**Authors:** Carlos F. M. Silva, Ana P. D. de M. S. Guerrinha, Sofia Carvalho, Diana C. G. A. Pinto, Artur M. S. Silva

**Affiliations:** Laboratório Associado para a Química Verde-Rede de Química e Tecnologia (LAQV-REQUIMTE), Department of Chemistry, University of Aveiro, 3810-193 Aveiro, Portugal; silva.c@ua.pt (C.F.M.S.); patriciaguerrinha@ua.pt (A.P.D.d.M.S.G.); sofiapereiracarvalho@ua.pt (S.C.); diana@ua.pt (D.C.G.A.P.)

**Keywords:** Alzheimer’s disease, acetylcholinesterase, amyloid-*β*, BACE-1, 1,3,5-triazines, MTDLs

## Abstract

Currently, Alzheimer’s disease (AD) is one of the most frequent forms of dementia. From a molecular perspective, the molecular characteristics that better define this disease consist of abnormal protein deposits between neuronal cells, namely senile plaques (SPs) and neurofibrillary tangles (NFTs), consisting of protein aggregates of amyloid-*β* and hyperphosphorylated tau protein, respectively. In addition to these protein aggregates, a third molecular hallmark of AD consists of depleted neurotransmitter acetylcholine levels. To date, the treatments developed for this disease are mostly focused on the use of AChE inhibitors, presenting only a symptomatic approach against the disease instead of a cure. Triazines are nitrogen-containing heterocyclic compounds that, throughout the years, have attracted a lot of curiosity from medicinal chemists for presenting numerous biological properties and being widely present in nature. In particular, this class of compounds has been associated with inhibiting several biological targets, emerging as a promising class for developing new pharmacological agents. However, there is still a scarcity of knowledge regarding the potential of this type of compound against any of the hallmarks of AD. For this reason, this paper intends to fulfill this absence by highlighting the potential of a subclass of triazines, 1,3,5-triazines (*sym*-triazines), as promising molecules for developing novel AD treatments. Thus, an in-depth analysis of 1,3,5-triazine derivatives is performed regarding its inhibitory activity against AChE (cholinergic hypothesis) and its capability to inhibit amyloid-*β* formation and aggregation (amyloid hypothesis). Through this analysis, it is possible to indicate some structural features optimal for each described activity, a compilation that we believe to be essential for the scientific community in this never-ending pursuit.

## 1. Introduction

**Figure 1:**
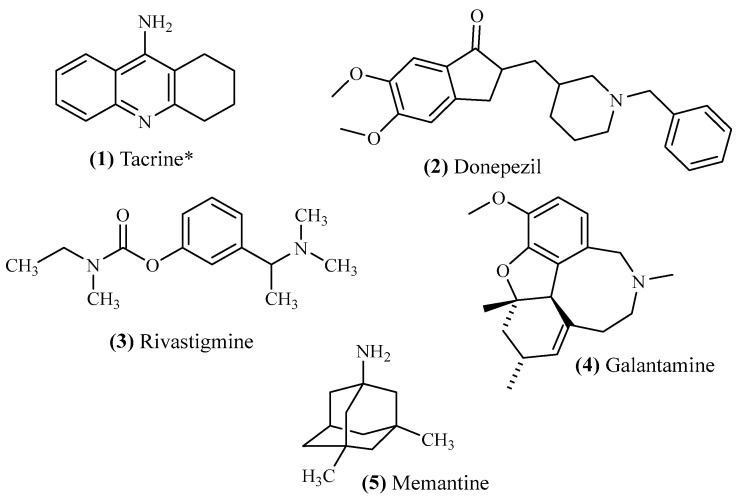
Drugs with FDA approval for the treatment of AD. (* *Discontinued*).

Dementia is a syndrome of chronic and progressive nature that causes the deterioration of the cognitive system, mainly affecting memory, thinking, emotional control, and social behavior [1]. One of the most common varieties of dementia (around 60–70%) is Alzheimer’s disease (AD), which, until 2016, was also one of the 10 main global causes of death (Figure 1). Statistically, Alzheimer’s Disease International indicates that every 3 s, an individual develops dementia in the world. From a worldwide perspective, now, 50 million people are estimated to suffer from this condition. These numbers are expected to rise to 82 million and 152 million in 2030 and 2050, respectively, which suggests an increase of 10 million cases each year [2].

AD consists of an irreversible and incurable progressive neurodegenerative disorder that first affects the patient’s brain function, including neuronal deterioration and consequent neuronal death, and significant loss of cholinergic transmission [3]. However, numerous mechanisms, such as inflammatory, biochemical, metabolic, and oxidative processes, have also been associated with the development of the disease and its multifactorial side. Although there is still a lot to uncover about AD, some risk factors have been reported throughout the years, such as age, the significant risk factor, genetic factors, the environment or even associated conditions like diabetes or cardiovascular issues [4,5,6].

Currently, dealing with AD continues to be directed towards the delay and control of the symptoms rather than its cure. So far, five drugs have been approved by the Food and Drug Administration (FDA): tacrine (**1**) (1993–2013), donepezil (**2**) (1996), rivastigmine (**3**) (2000), galantamine (**4**) (2001), and memantine (**5**) (2003) (Figure 1) [7]. Tacrine (**1**) was the first acetylcholinesterase inhibitor (AChEI) approved for the treatment of AD, in 1997, but showed limitations due to hepatotoxic side effects and was later withdrawn in 2013 [8]. Donepezil (**2**), rivastigmine (**3**), and galantamine (**4**) were later followed as acetylcholinesterase inhibitors, showing similar efficacy [9]. In turn, memantine (**5**) is a non-competitive *N*-methyl-D-aspartate receptor (NMDAR) antagonist with neuroprotective and anti-inflammatory effects that prevents neuron loss and improves symptoms by helping restore the function of damaged neurons [10]. Even though some drugs have already been developed against this disease, the development of novel treatments still faces numerous challenges and limitations. The main challenge continues to be the complexity and multifactorial nature of AD, with multiple metabolic pathways being associated with the disease’s development and progression. In addition, most of the developed compounds targeting AD struggle to cross the blood–brain barrier (BBB), hampering drug delivery and making these molecules impossible to be used to treat this disease.

### 1.1. Alzheimer’s Disease

**Figure 2:**
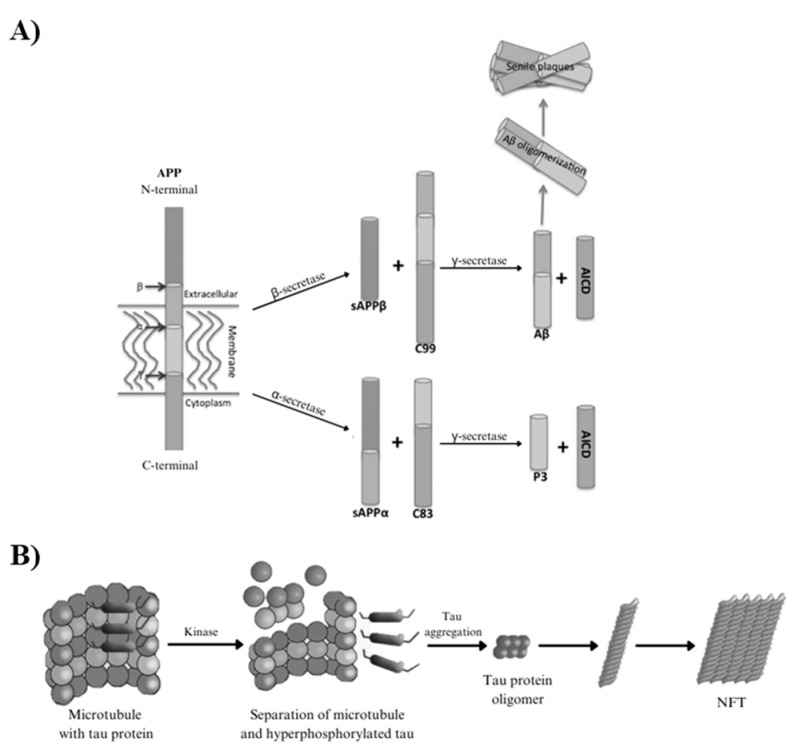
(**A**) Formation of senile plaques from APP. (Adapted from Sun et al. [17]): The insoluble A*β* fibrils are formed when BACE-1 replaces α-secretase in the cleavage of APP, leading to altered neurotransmission, neuroinflammation, brain oxidative stress, and synaptic dysfunction; and (**B**) formation of neurofibrillary tangles. Adapted from Šimic et al. [18]: The hyperphosphorylation of tau (*τ*) protein leads to a decrease in the τ protein’s affinity for microtubules, and consequent increase in cytosolic *τ* protein, causing aggregation into oligomers and aggregates and microtubule destabilization.

From a molecular point of view, AD is characterized by several pathological markers in the brain’s central nervous system (CNS), namely synaptic dysfunction and abnormal deposits of proteins: senile plaques (SPs), and extracellular deposits of insoluble amyloid-*β* (A*β*) fibrils, and neurofibrillary tangles (NFTs) [11,12]. The insoluble A*β* fibrils are formed when *β*-secretase (BACE-1) substitutes α-secretase in the accurate cleavage of the Amyloid Precursor Protein (APP), a protein responsible for the regulation of the neuronal cells, contributing to their growth and regeneration, leading to altered neurotransmission, neuroinflammation, brain oxidative stress, and synaptic dysfunction (Figure 2) [13,14]. Conversely, NFTs are intracellular filamentous inclusions composed of an abnormally aggregated and hyperphosphorylated tau (*τ*) protein. This protein plays a vital role in regulating and assembling neuronal microtubes and in the movement of organelles along axons and dendrites (Figure 2) [15,16].

Another characteristic of AD consists of the loss of cholinergic neurons and the reduction in acetylcholine (ACh) levels, an important neurotransmitter in the brain, which is related to the capacity of learning, memory and emotional behavior in the brain, the areas more affected in AD [19]. This reduction in the levels of ACh may be associated with both a reduced production of the neurotransmitter and an exacerbated activity of the enzyme acetylcholinesterase.

Even though the mechanisms of the disease remain mostly unclear, various hypotheses have already been suggested and discussed in the scientific community, of which the “*Cholinergic hypothesis*” [20] and “*Amyloid cascade hypothesis*” [21] are the most accepted in the scientific world. With AD as a multifactorial disease, these hypotheses try to explain how AD develops and which molecular phenomenon triggers the cascade of effects characteristic of the disease. Even though a consensus is still to be achieved amongst the scientific community, the main molecular features associated with each of these hypotheses, namely the decrease in cholinergic levels (“*Cholinergic hypothesis*”) and A*β* aggregation (“*Amyloid cascade hypothesis*”), remain as the main focus for the development of novel treatments for AD.

### 1.2. Multitarget in Alzheimer Disease

In the 20th century, the predominant strategy for drug development was derived from the doctrine “one molecule, one target, one disease”. This strategy thrived on identifying and optimizing small molecules that act on a specific target, believed to be the only one responsible for the disease [22]. However, this approach started to change with the growing awareness that, for multifactorial diseases such as cancer, neurodegenerative diseases, cardiovascular diseases, or inflammation, the single-target drugs present unsatisfactory efficacy. As a response, developing novel multitarget therapeutic strategies to achieve higher effectiveness and safety while overcoming drug resistance followed two main approaches: the mixture of different monotherapies and the development of multitarget-directed ligands (MTDLs). While the first approach focuses on drug cocktails, raising various concerns regarding adverse effects, drug–drug interactions and poor patient compliance, MTDL usually consists of a single hybrid molecule containing two or more active pharmacophores, avoiding the limitations associated with the first approach [23,24]. Thus, the efforts to design and develop multifunctional drugs that can fight against this disease have increased in the scientific community, particularly by medicinal chemistry research groups [25,26]. This approach’s potential has been widely corroborated, as its implementation has demonstrated considerable results for other multifactorial diseases, such as HIV-1 [23], cancer [27], depression [28], and schizophrenia [29].

Regarding AD, numerous molecules have also been developed as MTDLs, being able to act as enzyme inhibitors and receptor ligands towards several targets, including cholinesterase enzymes (AChE and BuChE), A*β* peptides, hyperphosphorylated τ protein, amongst others [30]. In addition, some scaffolds and pharmacophores that, until recently, have only been studied for one particular target might have the potential to be further developed as MTDL. In this paper, we intend to address the different 1,3,5-triazine derivatives capable of acting against AD development by inhibiting AChE, A*β* production, and aggregation or even by acting as multitarget-directed ligands (MTDLs). Furthermore, we also intend to focus on the compilation of structural information essential to the scientific community for the development of novel therapies against this multifactorial disease.

## 2. 1,3,5-Triazines: A Promising Scaffold Against Alzheimer’s Disease

**Figure 3:**
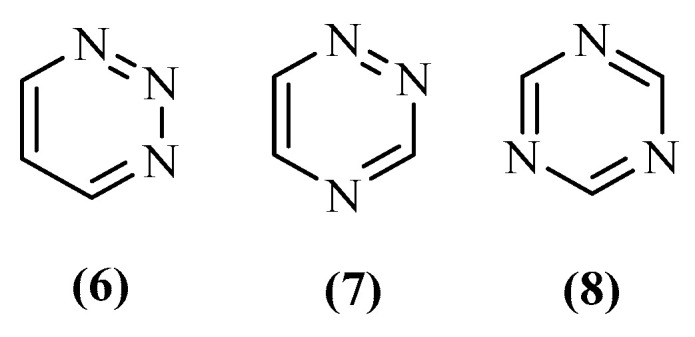
Structures of the triazine subclasses: 1,2,3-triazines (**6**), 1,2,4-triazines (**7**) and 1,3,5-triazines (**8**).

In the past decades, medicinal chemistry has made enormous progress in developing new compounds with promising pharmacological activities, which is closely related to the efforts of medicinal chemists. Heterocyclic compounds, especially *N*-containing heterocyclic compounds, have been a major focus of study in organic chemistry due to their abundance in nature and importance in several industries, including the pharmaceutical industry, in which they serve as constituents for drug design [31]. Among the *N*-containing heterocyclic compounds, triazines have been one of the most studied scaffolds, especially due to their unique structure composed of a benzyl ring with three carbon atoms replaced by nitrogen atoms (Figure 3) [32,33,34,35]. Structurally, the major differences between a triazine ring and a benzene ring are essentially related to the nitrogen atoms that increase the electron density and may, consequently, destroy the symmetry. This asymmetry can cause impairment of stability and promote the implementation of substitution and addition reactions. This family of compounds is composed of three distinct subclasses distinguished by the position of the nitrogen atoms, namely 1,2,3-triazines (**6**), 1,2,4-triazines (**7**) and 1,3,5-triazines (**8**) (Figure 3). These characteristics allow the triazines to be reported as having a huge variety of biological activities, such as antimalarial, antiviral, anti-inflammatory, antitumoral, and antimicrobial. They can even be used as dendrimers for drug delivery systems [33,36].

With respect to AD, the triazine scaffold has been described as possessing a broad variety of biological activities, including a neuroprotective effect, combined with a potential inhibitory effect against AChE and the ability to intercalate between A*β* sheets, promoting its disaggregation [37,38]. These properties, combined with the virtually unlimited range of potential structural modifications that can be performed, leave room for the design and development of new drugs for the treatment of numerous diseases, particularly 1,3,5-triazine derivatives.

### 1,3,5-Triazines in a Multitarget Model for AD

**Figure 4:**
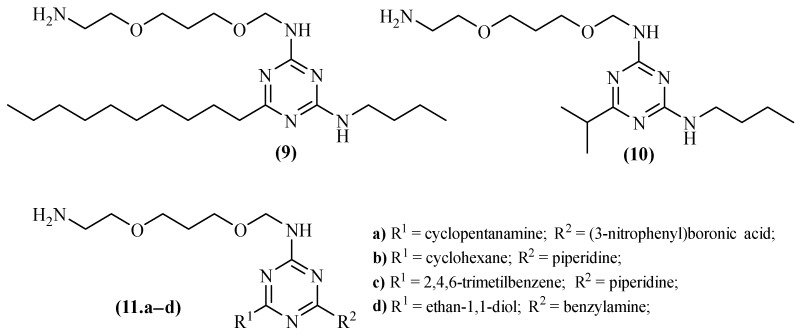
Structure of AA3E2 (**9**), AA3D2 (**10**) and analogs, 1,3,5-triazines with promising inhibitory properties against A*β* fibril formation.

In 2006, through a high-throughput study, two particular triazine derivatives were identified as being able to inhibit A*β* fibril formation, namely AA3E2 (**9**) and AA3D2 (**10**), with the results demonstrating that AA3E2 (**9**) shows better inhibition of A*β* aggregation than AA3D2 (**10**) (Figure 4) [39]. Four years later, in 2010, a different research group further examined the main differences in the interactions between both compounds **9** and **10** and the senile plaques in a study essentially based on its structure [40]. The results demonstrated that AA3D2 (**9**) is a better inhibitor of A*β* aggregation, mostly due to its interaction with the central hydrophobic region of the protein. In this case, the hydrocarbon chain of the compound interacts with two phenylalanine residues, Phe19 and Phe20. Additionally, three H-bonds are formed between the AA3E2 (**9**) and another protein residue, Glu22, which might stabilize the compound–protein interaction. On the other hand, mainly due to its reduced hydrocarbon chain, AA3D2 (**10**) cannot interact with Phe19 and Phe20, proving that a bigger hydrocarbon chain allows better inhibition through interactions with the protein’s residues. Influenced by these results, in 2008, another series of compounds was evaluated for its potential of inhibiting A*β* aggregation, compounds that maintain an *N*^2^-((3-(2-aminoethoxy)propoxy)methyl) of the previous research work while variating the other two other substituents [41]. Through a high-throughput screening assay, some compounds were revealed to be promising inhibitors of A*β* aggregation (**11.a–d**), Figure 4. Particularly, two compounds, namely *N*-((3-(2-aminoethoxy)propoxy)methyl)-4-cyclohexyl-6-(piperidin-1-yl)-1,3,5-triazin-2-amine (**11.a**) and *N*-((3-(2-aminoethoxy)propoxy)methyl)-4-mesityl-6-(piperidin-1-yl)-1,3,5-triazin-2-amine (**11.b**), demonstrated the higher values of inhibition. Furthermore, comparing compounds with similar substitution patterns made it clear that the presence of steric bulk substituent groups in both positions might be an important structural feature for this particular activity.

**Figure 5:**
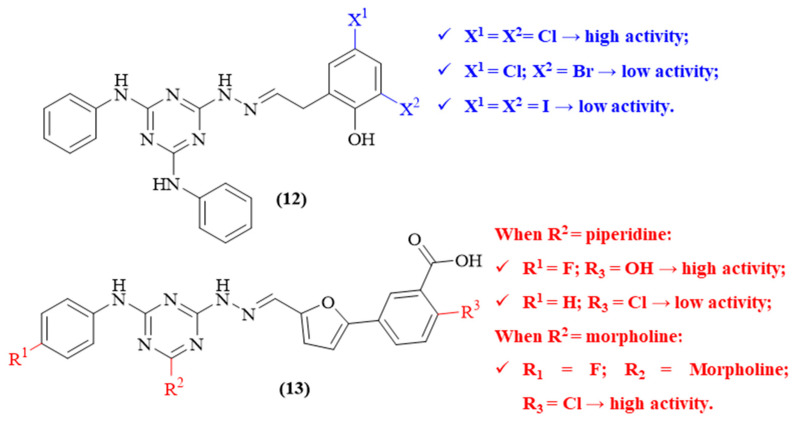
BACE-1 inhibitors containing 1,3,5-triazine scaffold (**12** and **13**).

Still in 2006, Huang and colleagues reported a study where an in silico high-throughput screening was employed for the discovery of potential non-peptide inhibitors of BACE-1, allowing the theoretical evaluation of more than 300,000 small molecules [42]. From this library of molecules, eighty-eight compounds were evaluated through an in vitro BACE-1 enzymatic assay. The results allowed for identifying ten active molecules (IC_50_ < 100 µM) that, interestingly, shared a 1,3,5-triazine scaffold. Structurally, these ten molecules can be divided into two groups (**12** and **13**), based on the substituent groups common to each other. Considering the series of compounds **12** (Figure 5), the substitution pattern of the aromatic ring from the hydrazone fragment appears to considerably affect the compound’s BACE-1 inhibition, with low-molecular-weight halogens promoting higher inhibition levels than with higher molecular weight halogens (IC_50_ 11.2 ± 0.2 μΜ and IC_50_ 25.5 ± 8.4 μM). Additionally, considering the second group of derivatives (**13**), it was observed that the presence of 2-hydroxybenzoic acid, piperidine, and fluorobenzene (**13.a**) promote a good activity (IC_50_ 27.9 μM), since these fragments occupy some of the enzyme’s pockets (S2, S2′ and S3′), while the presence of chloro and hydrogen atoms (**13.b**) promotes a lower inhibition capability (IC_50_ 151.8 μM). Finally, the conjugation of a fluorine atom as R^1^, morpholine as R^2^ and a chloro atom as R^3^ (**13.c**) appears to be the best substitution pattern for this type of compound, creating the most active derivative (IC_50_ 7.1 μM).

**Figure 6:**
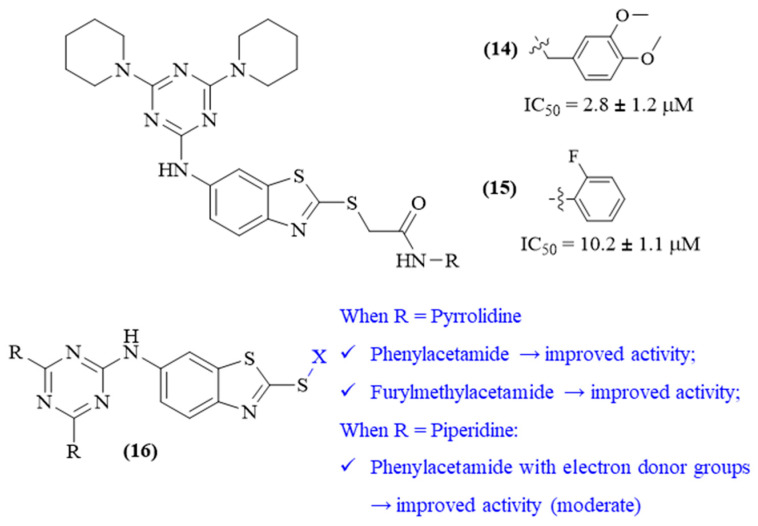
Benzothiazole-containing 1,3,5-triazines with the capacity to inhibit the BACE-1. The color blue intends to highlight the X substituent group.

In 2009, a research work focused on developing BACE-1 inhibitors as potential agents for the treatment of AD, following the suggestion that the inhibition of this particular enzyme may be crucial to combat this brain disease. This work led to the discovery of two 1,3,5-triazine derivatives containing a benzothiazole fragment (**14**), with good levels of BACE-1 inhibition, amongst several other non-peptide BACE-1 inhibitors (Figure 6) [43]. Based on this previous work, another interesting research was performed with the purpose of developing a complete structure–activity relationship study of this particular type of 1,3,5-triazine derivative containing a benzothiazole fragment in one of its positions (**15**), Figure 6 [44]. This basic structure appears to be beneficial for the evaluated activity since the interaction between the NH of the benzothiazole fragment and the Asp32 of the enzyme, through an H-bond, is possible and indispensable for the inhibition. The remaining two substitutions varied from pyrrolidinyl to piperidinyl and morpholinyl groups. By solely considering these two substitutions, the presence of two pyrrolidinyl fragments seems to create derivatives with better inhibition (0.12 μM < IC_50_ < 20 μM), followed by the introduction of two piperidinyl fragments with moderate activity (17.2 μM < IC_50_ < 50 μM), and two morpholine fragments with almost no activity for the majority of compounds synthesized (14.7 μM < IC_50_ < 50 μM). Finally, the benzothiazole fragment was also modified, with different substituent groups being introduced at the X position to assess the influence of this particular position on its BACE-1 inhibitory activity.

Regarding the pyrrolidinylic derivatives, those containing phenylacetamide groups demonstrated improved activity levels against BACE-1, with a preference for shorter distances between the amide group and the phenyl ring (IC_50_ 0.12 μΜ) when compared with longer distances (IC_50_ 1.7 μΜ). In addition, the introduction of a furyl methyl acetamide in this particular position also promotes an increase in the activity (IC_50_ 0.69 μΜ). Finally, the presence of an amide or ester group between the benzothiazole fragment and the phenyl ring appears to be crucial for these compounds’ inhibitory properties, since its absence leads to a derivative with considerably lower activity levels (IC_50_ ~20 μΜ). Considering the piperidinylic compounds, the presence of an electron donor group, like a methoxy group, or a fluorine atom in the phenylacetamide fragment, seems to improve the inhibitory activity of these compounds towards BACE-1. In relation to the morpholinic compounds, except for one derivative that presented some level of activity (IC_50_ 14.7 μM), the remaining derivatives were all practically inactive.

**Figure 7:**
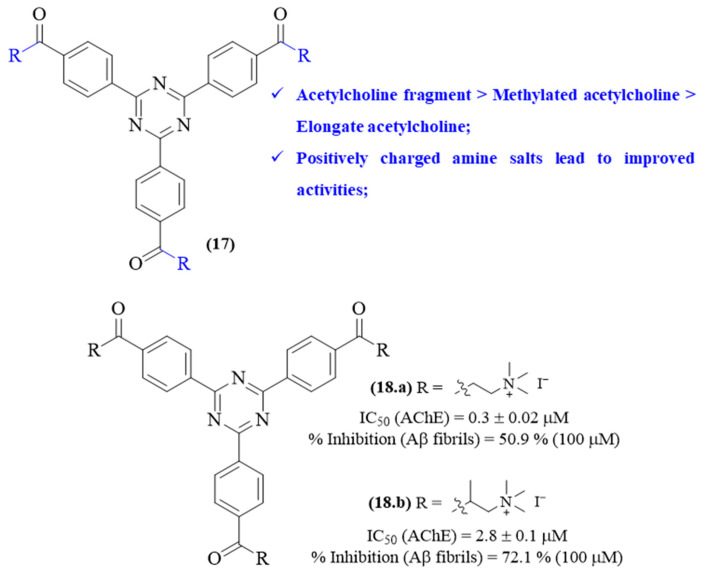
Structure–activity relationship study of acetylcholine-containing *sym*-triazines (**17**) with particular emphasis to derivatives **18.a** and **18.b**.

In 2013, Veloso and colleagues synthesized a series of *sym*-triazines containing acetylcholine-like substituents with aromatic phenyl rings and evaluated them for their ChE-inhibitory activity and capability to modulate A*β* aggregation (Figure 7) [45]. This research work demonstrated that some of these compounds present considerable AChE-inhibitory levels associated with AChE/BuChE selectivity and mixed-type inhibition mechanisms. Furthermore, most of the derivatives were also shown to effectively inhibit the A*β* fibril formation, presenting higher inhibition levels than the positive control (peptide iA*β*5p). Structurally, considering the inhibition of AChE, it became clear that the increased incorporation of acetylcholine-like substituent groups led to improved AChE-inhibitory activities, with the most active derivatives containing three acetylcholine-like groups. Furthermore, three acetylcholine-like substituent groups were evaluated: the exact acetylcholine fragment, acetylcholine with an elongated alkyl chain and acetylcholine with an additional methyl group. The introduction of these different acetylcholine-type groups also led to distinct levels of inhibition, with the exact acetylcholine fragment being the most effective, followed by the acetylcholine with an additional methyl and acetylcholine with elongated alkyl chain. Finally, it is also possible to verify that the conversion of these derivatives into positively charged amine iodine salts considerably contributes to improved inhibitory activity levels. Considering its A*β* aggregation inhibition, similar to the AChE inhibition, it became clear that the number of acetylcholine-like fragments introduced to the 1,3,5-triazine core affects the derivatives’ ability to inhibit A*β* aggregation, with the introduction of three acetylcholine-like substituent groups being the most effective. Interestingly, in this case, the additional methyl group in the acetylcholine fragment seems to benefit the inhibition of A*β* aggregation, suggesting that the increased hydrophobicity of these derivatives might play a crucial role in this effect. In the same year, this same research group was able to demonstrate that the two most active derivatives (**17** and **18**) are not only able to effectively inhibit AChE and A*β* aggregation but also promote several beneficial effects on human neurons, including the stimulation of neuronal cellular process length and branching, increasing expression of synaptophysin, a synaptic marker protein, and upregulation of MAP2, a neuronal differentiation marker [46].

**Figure 8:**
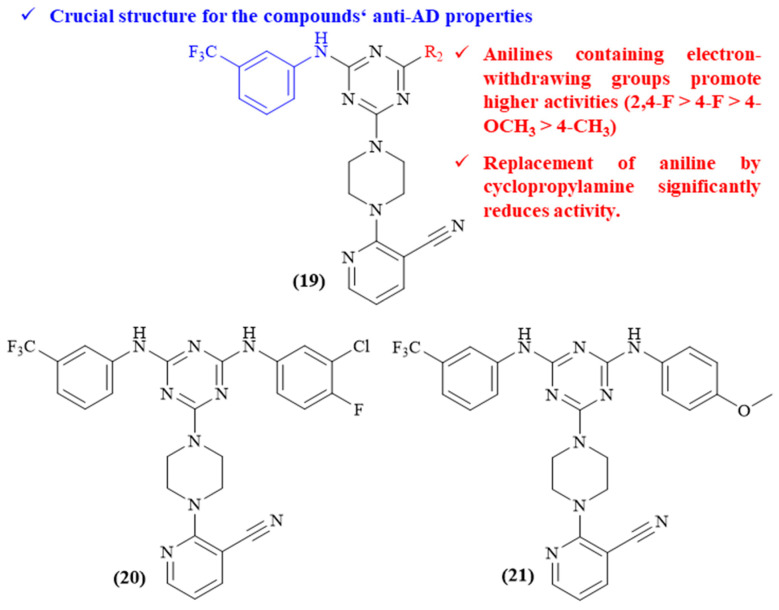
Structure–activity relationship study of novel cyanopyridine–triazine hybrids (**19**), with particular emphasis to the two most promising compounds from the series (**20** and **21**). The different colors intend to individualize the different substituent groups of the molecule.

Three years later, in 2016, Maqbool and colleagues focused their efforts on the development of a series of eight novel cyanopyridine–triazine hybrids and evaluated their potential as MTDLs against AD (Figure 8) [47]. These compounds were characterized by containing a 1,3,5-triazine scaffold substituted by a 3-cyanopyridine linked through a piperazine and two variable anilines (or in one case, an aniline and cyclopropylamine). To assess the multitarget potential of these compounds, several properties were evaluated, including their inhibitory activity against ChEs, A*β* disaggregation, oxidative stress, cytotoxicity, and neuroprotection against A*β*-induced toxicity. However, even though some of these compounds present promising effects for all the evaluated activities, this analysis will be mainly focused on their effects against two of AD’s hallmarks, ChEs’ inhibition and A*β* disaggregation. Structurally, considering the inhibition of AChE, it is possible to verify that the presence of a 3-(trifluoromethyl)aniline as one of the substitutions appears to be crucial for the inhibition of AChE, since its replacement leads to a considerable decrease on the compounds’ activity (IC_50_ from 0.153 ± 0.01 and 0.080 ± 0.005 μΜ to 1.701 ± 0.08 and 1.412 ± 0.02 μΜ). Furthermore, the substitution pattern of the introduced anilines also seems to considerably influence the compounds’ properties, with electron-withdrawing substituent groups leading to more active derivatives (2,4-F > 4-F > 4-OCH_3_ > 4-CH_3_). Finally, the replacement of one of the aniline fragments by a cyclopropylamine significantly reduces the potency of these derivatives (IC_50_ 0.528 ± 0.04 μΜ). Regarding the potential of this series against A*β* aggregation, the results demonstrated that the triazine core allowed the compounds to directly inhibit A*β* aggregation through intercalation between *β*-amyloid sheets. Interestingly, the most active derivatives against A*β* aggregation correspond to the most active derivatives against AChE, which might suggest that these compounds can also indirectly influence A*β* aggregation by interacting with the PAS site of AChE, something that is corroborated by the molecular docking studies.

**Figure 9:**
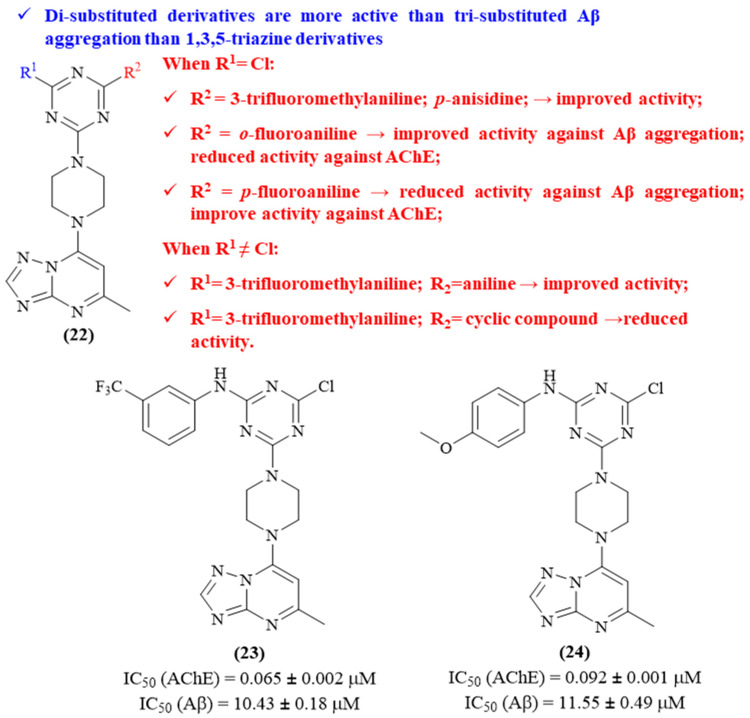
Structure–activity relationship study of novel 1,3,5-triazine-triazolopyrimidine hybrids (**22**), with particular emphasis on the two most promising compounds from the series (**23** and **24**). The different colors intend to individualize the different substituent groups of the molecule.

In the next year, the same research group decided to explore a little further the use of 1,3,5-triazine derivatives for the development of MTDLs against AD, with the synthesis of triazine–triazolopyrimidine hybrids being the main focus of this new work (Figure 9) [48]. Besides this fragment, the remaining substitutions were considerably variable, contemplating a 3-(trifluoromethyl)aniline, *p*-anisidine, *p*-fluoroaniline, *o*-fluoroaniline, and 3-chloro-4-fluoroaniline, amongst other amine groups (**22**, Figure 9). The results demonstrated that, in general, disubstituted 1,3,5-triazine derivatives are better AChE inhibitors than trisubstituted 1,3,5-triazine derivatives. Furthermore, the introduction of a 3-(trifluoromethyl)aniline promotes increased AChE inhibition (IC_50_ 0.065 ± 0.002 μM), followed by the introduction of a *p*-anisidine (IC_50_ 0.092 ± 0.001 μM). Additionally, by comparing the functionality of *p*-fluoroaniline and *o*-fluoroaniline, it becomes clear that the *p*-position promotes higher inhibition levels (IC_50_ 0.115 ± 0.11 μΜ) than the *o*-position (IC_50_ 0.286 ± 0.52 μM). Finally, the planar structure of aromatic groups might also have a great influence towards this activity, since their replacement by another cyclic group produces less active derivatives (IC_50_ from 0.388 ± 0.19 μΜ to 1.25 ± 0.33 μΜ). Considering its potential against A*β* aggregation, once again, it was possible to verify that disubstituted 1,3,5-triazine derivatives are better at A*β*-aggregation than trisubstituted 1,3,5-triazine derivatives. Interestingly, the two most active derivatives against A*β*-aggregation correspond to the most active derivatives against AChE, containing 3-trifluoromethylaniline and *p*-anisidine as substituent groups (IC_50_ 10.43 ± 0.18 μΜ and IC_50_ 11.55 ± 0.49 μΜ, respectively). However, through a more profound analysis, it is possible to observe that the disubstituted derivatives containing *p*-fluoroaniline and *o*-fluoroaniline as substituent groups exhibit different inhibition levels, the *o*-structure being better for inhibiting A*β*-aggregation. As verified for the inhibition of AChE, the replacement of any aniline by another cyclic group produces less active derivatives, even when conjugated with 3-(trifluoromethyl)aniline (IC_50_ > 25.00 μΜ).

**Figure 10:**
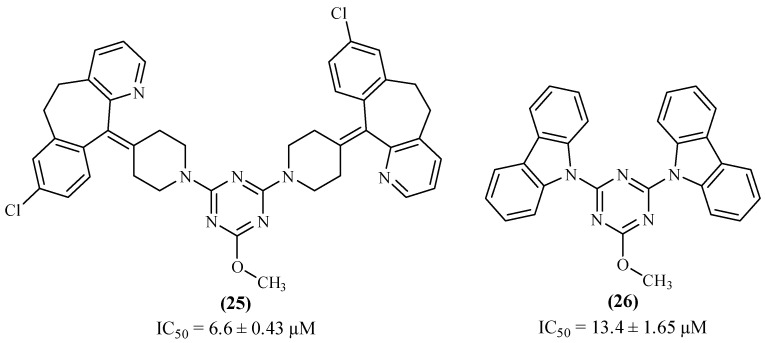
1,3,5-triazine derivatives containing desloratadine fragments (**25**) and carbazole fragments (**26**) as promising AChE inhibitors.

In 2019, another research group focused their efforts on the design, synthesis and evaluation of tricyclic fused ring systems as promising inhibitors of AChE. Amongst these derivatives, two symmetric 1,3,5-triazines (**25** and **26**) were synthesized and evaluated for its inhibitory activity against AChE, one containing desloratadine fragments (**25**) and another containing carbazole fragments (**26**) as substituent groups (Figure 10) [49]. The results demonstrated that derivative **25**, containing the desloratadine fragments, exhibits a better inhibition level (IC_50_ 6.6 ± 0.43 µM) when compared with compound **26**, containing carbazole fragments, a compound that only shows a moderate activity level (IC_50_ 13.4 ± 1.65 µM). This difference might be explained by the fact that the two desloratadine rings of derivative **25** interact with both catalytic sites (CAS and PAS), through ᴨ-ᴨ stacking, and the anionic site through hydrogen bonds. On the other hand, the two carbazole fragments of derivative **26** only have the capacity to interact with the catalytic site, thus not affecting the anionic site of AChE.

**Figure 11:**
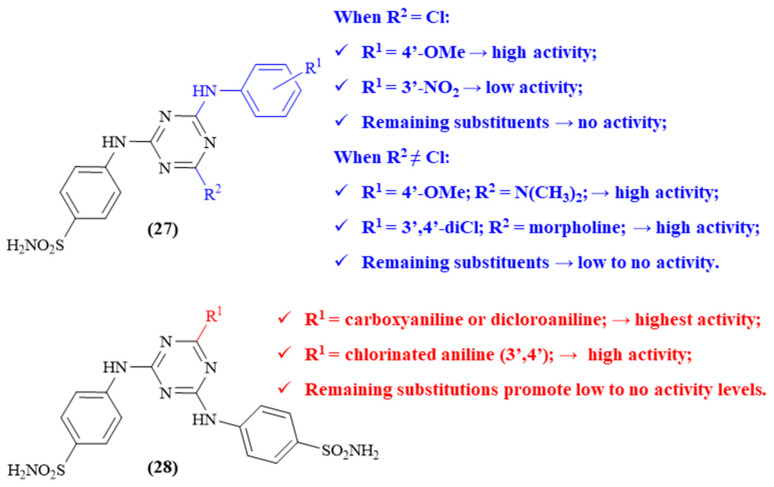
Structure–activity relationship of sulfonamide-containing 1,3,5-triazine derivatives (**27** and **28**). The different colors intend to individualize the different substituent groups of the molecule.

One year later, Lolak and colleagues proceeded to synthesize a series of benzenesulfonamides incorporating 1,3,5-triazines, with the remaining substitutions contemplating the introduction of substituted anilines, dimethylamine, morpholine or piperidine groups (**27**, Figure 11) [50]. This series of derivatives was then evaluated for their antioxidant properties and inhibitory activity towards AChE and BuChE. Even though all obtained compounds showed great BuChE-inhibitory activity, the same was not verified regarding AChE-inhibitory activity. Structurally, it was possible to verify that, for the disubstituted 1,3,5-triazines (R^2^ = Cl), the derivative containing a 4′-methoxyaniline presents the highest level of activity (96.37 ± 1.71% at 200µM), being even more active than galantamine (84.20 ± 0.74%). In turn, derivatives with anilines containing 4′-F, 3′,4′-diCl or 3′-NO_2_ present low to no AChE-inhibitory activity. Regarding the trisubstituted triazines, the combination of a 4′-methoxyaniline with a dimethylamine or a 3′,4′-dichloroaniline with morpholine demonstrates the higher levels of inhibition (91.10 ± 1.42% and 93.19 ± 2.33%, respectively, at 200 µM). The remaining substitution patterns present no significant values, with low to no inhibition levels (28.42 ± 0.76% at 200 µM).

Following their previous work, a new series of 1,3,5-triazine derivatives were synthesized by the same research group, consisting of a series of bis-sulfonamide-substituted 1,3,5-triazines (**28**, Figure 11) [51]. The remaining chlorine atom was then replaced by anilines, with different substitution patterns, or aliphatic amines to obtain enough diversity for the determination of an SAR study. The results demonstrated that the best substituent groups to promote the inhibition of AChE consist of the carboxyaniline or the dicloroaniline (IC_50_ 397.32 nM and 420.14 nM, respectively). Furthermore, it was possible to verify that the replacement of the aniline fragment by an aliphatic amine leads to weaker inhibitors, suggesting that the presence of an aromatic amine is essential for the compounds’ activity against AChE. Finally, the presence of chlorine atoms on the aniline fragment also appear to promote increased levels of AChE inhibition, with two adjacent chlorine atoms promoting even higher activity levels (IC_50_ from 502.15 nM to 420.14 nM). In addition to the activity evaluation, some theoretical studies were also performed to evaluate the compounds’ interactions with the enzyme, from which it was possible to observe that the 1,3,5-triazine–enzyme interaction might be possible by H-bonds with Tyr124, Leu289, His287, and Ser293 and ᴨ-ᴨ stacking interactions with Trp286.

**Figure 12:**
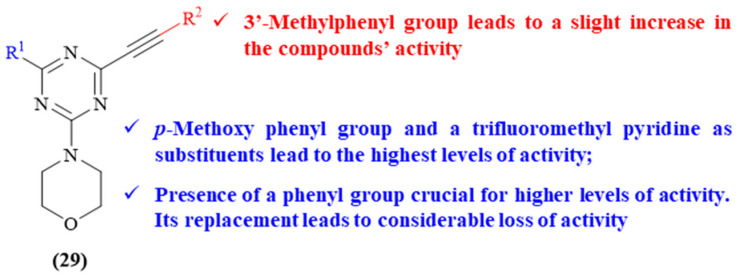
Structure–activity relationship of morpholine-containing 1,3,5-triazine derivatives (**29**). The different colors intend to individualize the different substituent groups of the molecule.

In 2021, Reddy and colleagues developed a series of eight novel 1,3,5-triazine derivatives containing a morpholine fragment as a substituent and evaluated them for their antioxidant and ChE-inhibitory properties (**29**, Figure 12) [52]. The results demonstrated that only four of the derivatives present considerable levels of AChE-inhibitory activity, with IC_50_ values from 8.49 ± 0.01 to 14.49 ± 0.05 μM. The most active derivatives consisted of those bearing a *p*-methoxyphenyl group (IC_50_ 10.02 ± 0.40 μM) and a trifluoromethyl pyridine (IC_50_ 8.49 ± 0.01 μM) as substituents. Structurally, it was possible to verify that the introduction of a methyl group on the alkyne phenyl group leads to a slight increase in the compounds’ activity (IC_50_ values from 14.49 ± 0.05 μM and 24.17 ± 4.12 μM to 12.38 ± 0.51 μM and 19.26 ± 1.88 μM, respectively). Furthermore, except for the pyridine ring, the presence of a phenyl group as the third fragment (R^1^) seems to be crucial for higher levels of activity, since its replacement for other aromatic systems or a simple methyl group leads to a considerable loss of activity. Through further studies, the authors demonstrated that these derivatives present a mixed-type inhibition and that their inhibitory properties are closely related with their capability to interact with both PAS and CAS sites of AChE, through H-bonds and ᴨ-ᴨ stacking interactions.

**Figure 13:**
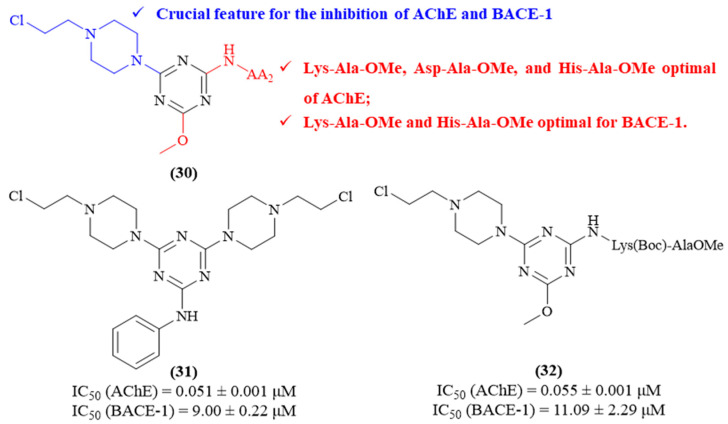
Structure–activity relationship of nitrogen mustard analogs containing the 1,3,5-triazine scaffold (**30**), with emphasis on the two most active derivatives (**31** and **32**). The different colors intend to individualize the different substituent groups of the molecule.

Still in 2021, another research group described the synthesis and evaluation of a series of nitrogen mustard analogs containing the 1,3,5-triazine scaffold and dipeptide residues, and evaluated them as potential inhibitors of both AChE and BACE-1, two of the main hallmarks of AD (**30**, Figure 13) [53]. The results of this work demonstrated that all the synthesized derivatives present significant inhibitory activities against both AChE and BACE-1, with derivatives **31** (IC_50_-_AChE_ 0.051 ± 0.001 μM and IC_50-BACE-1_ 9.00 ± 0.22 μM) and **32** (IC_50-AChE_ 0.055 ± 0.001 μM and IC_50-BACE-1_ 11.09 ± 2.29 μM) being the most active against both enzymes. Structurally, the relevance of the 2-chloroethyloamino fragment seems to be corroborated by its presence in the most active compound against both enzymes. Furthermore, the fragments Lys-Ala-OMe, Asp-Ala-OMe, and His-Ala-OMe seem to be optimal dipeptides to be introduced into the 1,3,5-triazine scaffold to promote higher AChE-inhibitory properties, while the fragments Lys-Ala-OMe and His-Ala-OMe seem to be optimal to promote the inhibition of BACE-1.

**Figure 14:**
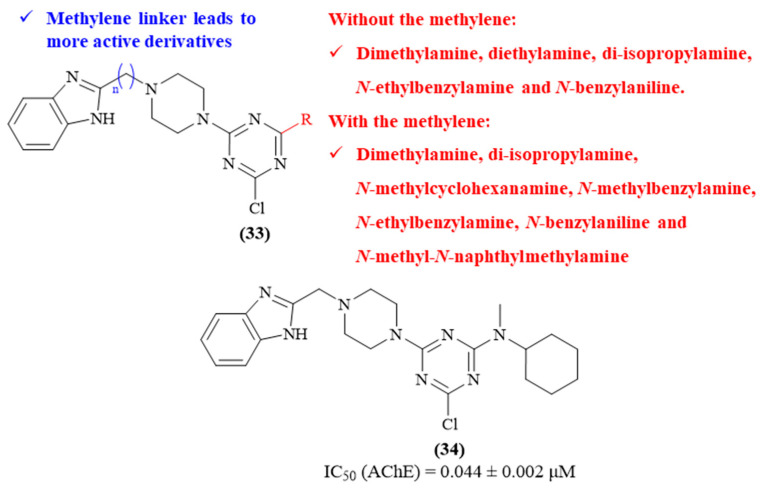
Structure–activity relationship of 1,3,5-triazine-benzimidazole hybrids (**33**), with emphasis on the most active derivative (**34**). The different colors intend to individualize the different substituent groups of the molecule.

One year later, in 2022, Wu and colleagues designed, synthesized and evaluated a series of twenty-four 1,3,5-triazine-benzimidazole hybrids as AChE inhibitors, based on the structure of donepezil (**33**, Figure 14) [54]. The results demonstrated that most of the twenty-four hybrids display AChE/BuChE selectivity, being considerably active against AChE and weak inhibitors against BuChE, with emphasis on compound **34** (IC_50_ 0.044 ± 0.002 μM) as the most active derivative of this series. Structurally, it was possible to verify that, in general, the presence of a methylene linker between the benzimidazole and the piperazine leads to more active derivatives. Furthermore, different substituent groups were introduced as a second introduction into the 1,3,5-triazine scaffold, allowing a deeper understanding of the influence of this addition, with different substituents promoting distinct effects depending on the presence or absence of the methylene group. Considering the compounds without the methylene, only five derivatives present promising AChE inhibition levels (IC_50_ < 10 μM), consisting of those bearing a dimethylamine, diethylamine, di-isopropylamine, *N*-ethylbenzylamine and *N*-benzylaniline. In turn, from those containing the methylene, eight derivatives present promising AChE inhibition levels (IC_50_ < 10 μM), consisting of those bearing a dimethylamine, di-isopropylamine, *N*-methylcyclohexanamine, *N*-methylbenzylamine, *N*-ethylbenzylamine, *N*-benzylaniline and *N*-methyl-*N*-naphthylmethylamine.

**Figure 15:**
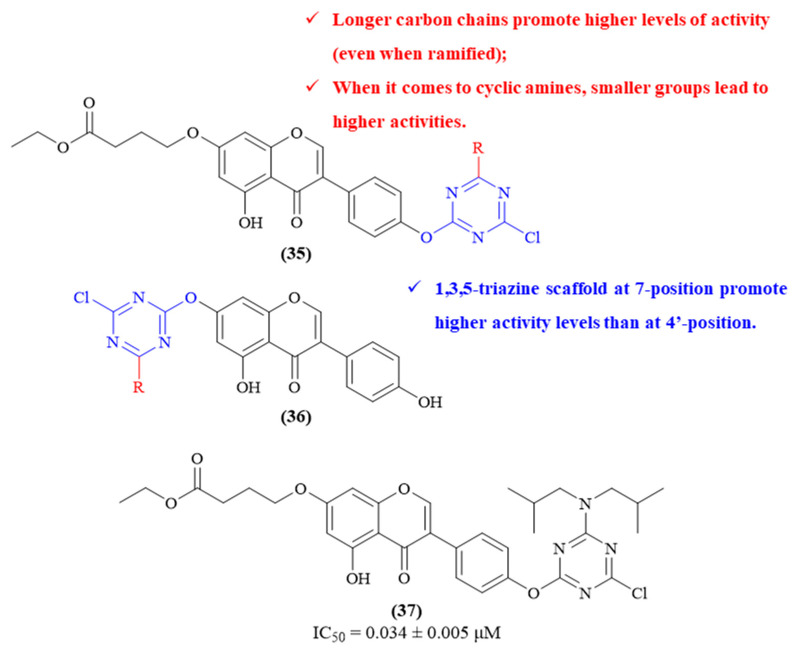
Structure–activity relationship of 1,3,5-triazine-genistein hybrids (**35** and **36**), with emphasis on the most active derivative (**37**). The different colors intend to individualize the different substituent groups of the molecule.

Based on their previous results (Figure 14), the same research group developed a series of twenty-four genistein-*O*-1,3,5-triazine derivatives and evaluated them as multifunctional anti-AD agents (**35** and **36**, Figure 15) [55]. The results demonstrated that most of these hybrids present considerable levels of activity, with emphasis on derivative **37** as the most active compound from the series (IC_50_ 0.034 ± 0.005 μM). Structurally, it was verified that the position where the 1,3,5-triazine scaffold is bonded to the genistein fragment has a considerable effect on the compounds’ AChE-inhibitory properties, with the group of compounds bearing the 1,3,5-triazine scaffold at the 7-position presenting higher activity levels than those at the 4′-position. Considering the most active group of derivatives (**36**), the different amino groups introduced into the 1,3,5-triazine fragment (R group) promoted distinct effects on the compounds’ activity. For instance, longer carbon chains seem to promote higher levels of activity (IC_50_ values from 1.34 ± 0.09 μM to 0.23 ± 0.03 μM), with the same happening for the ramified carbon chains (IC_50_ value from 5.73 ± 0.12 μM to 0.034 ± 0.005 μM). When it comes to cyclic amines, it becomes clear that the effect is the opposite, with the smaller cyclic amino groups leading to more active derivatives. Finally, regarding the most active compound from this series (**37**), the authors also described it as presenting a mixed-type inhibition mechanism, with molecular docking studies showing that this compound interacts with both CAS and PAS of the enzyme, similar to donepezil.

**Figure 16:**
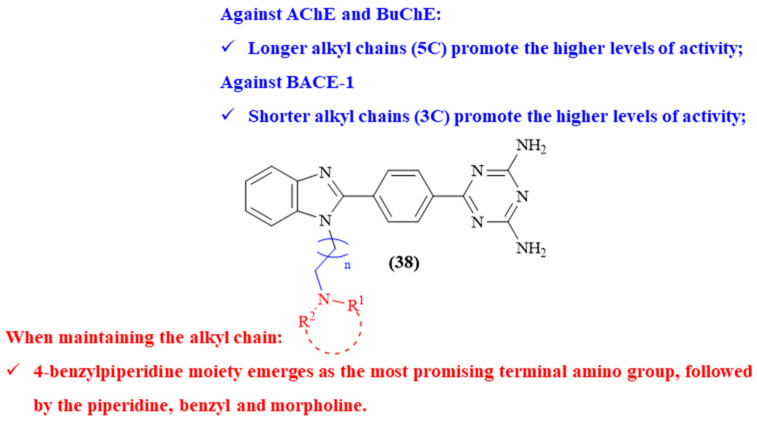
Structure–activity relationship of benzimidazole/1,3,5-triazine-2,4-diamine hybrids (**38**). The different colors intend to individualize the different substituent groups of the molecule.

Another research group focused on the design and synthesis of a series of twelve new benzimidazole/1,3,5-triazine-2,4-diamine hybrids (**38**, Figure 16) as potential multitarget agents for AD, evaluating their potential as inhibitors of AChE, BuChE and BACE-1 [56]. In this work, the results demonstrate that some of these derivatives present promising inhibitory activities against the studied enzymes. For instance, four of the evaluated derivatives present considerable levels of activity against AChE (IC_50_ values ranging from 19.01 ± 0.57 μM to 42.75 ± 0.38 μM), while seven display significant levels of activity against BuChE (IC_50_ values ranging from 11.23 ± 2.07 μM to 45.17 ± 1.65 μM). Regarding their activity against BACE-1, five molecules emerged as being active against the enzyme. Structurally, two main structural features were evaluated to understand its effect on the compounds’ inhibitory activity, namely the length of the alkyl chain between the benzimidazole and the terminal amino group, and the influence of the substitution pattern on the terminal amino group. Considering their potential against AChE, the results clearly demonstrate that longer alkyl chains bearing 5C promote higher levels of activity (IC_50_ 19.01 ± 0.57 μM; 21.09 ± 1.89 μM; 42.75 ± 0.38 μM; and 27.19 ± 1.05 μM), followed by 4C (IC_50_ > 100 μM) and 3C (IC_50_ > 100 μM). When maintaining the pentyl chain (5C), the 4-benzylpiperidine moiety emerged as the most promising terminal amino group (IC_50_ 19.01 ± 0.57 μM), followed by the piperidine (IC_50_ 21.09 ± 1.89 μM), the benzyl (IC_50_ 27.19 ± 1.05 μM) and morpholine (IC_50_ 42.75 ± 0.38 μM). When it comes to their activity against BACE-1, in contrast with the AChE inhibition, short alkyl chains (3C) promote the higher levels of activity, with 4-benzylpiperidine emerging as the optimal terminal amino group for this activity. Finally, the authors were also able to determine that the most active derivatives present a mixed-type inhibition for both AChE and BuChE, with molecular docking studies showing that these compounds interact with both enzymes. Regarding BACE-1, the theoretical studies showed that the most active compound binds to the key catalytic amino acid residues (ASP32 and ASP228) of the enzyme’s binding pocket.

**Figure 17:**
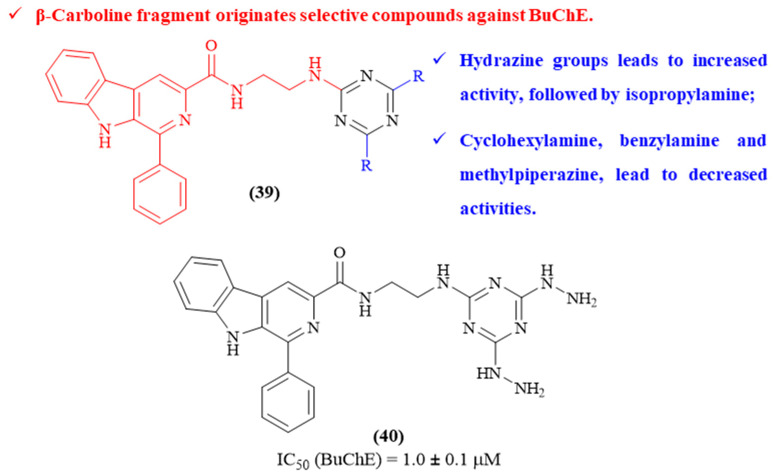
Structure–activity relationship of *β*-carboline-1,3,5-triazine hybrids (**39**) against BuChE, with emphasis on derivative **40** as the most active compound from this series. The different colors intend to individualize the different substituent groups of the molecule.

Still in 2022, Baréa and colleagues designed a series of five *β*-carboline-1,3,5-triazine hybrids and evaluated them for their AChE- and BuChE-inhibitory activities (**39**, Figure 17) [57]. The results demonstrated that the evaluated molecules display AChE/BuChE selectivity, presenting considerable inhibitory activity against BuChE (IC_50_ values ranging from 1.0 ± 0.1 μM to 18.8 ± 3.8 μM) and low activity against AChE (IC_50_ 100 μM). Structurally, these results demonstrate that the introduction of a *β*-carboline fragment produces compounds selective against BuChE, which can be explained by the larger BuChE active site gorge that allows the accommodation of these compounds, in contrast to AChE. Furthermore, considering the other two substitutions of the 1,3,5-triazine scaffold, the introduction of hydrazine groups leads to an increase in the compounds’ inhibitory activity (IC_50_ 1.0 ± 0.1 μM), followed by isopropylamine (IC_50_ 5.8 ± 4.1 μM). In turn, the remaining introductions to the 1,3,5-triazine scaffold, namely cyclohexylamine (IC_50_ > 100 μM), benzylamine (IC_50_ 12.0 ± 1.4 μM) and methylpiperazine (IC_50_ 18.8 ± 3.8 μM), lead to a decrease in activity compared to the chlorinated scaffold (IC_50_ 10.2 ± 0.3 μM).

**Figure 18:**
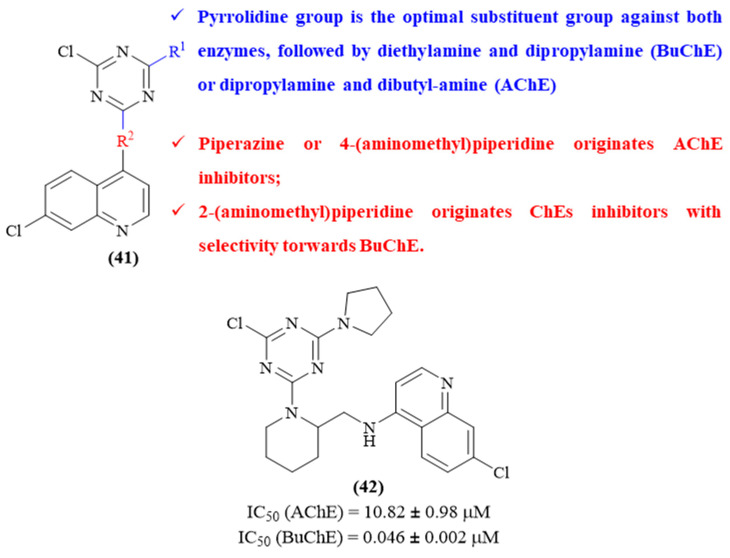
Structure–activity relationship of 1,3,5-triazine-quinoline hybrids (**41**) against BuChE, with emphasis on derivative **42** as the most active compound from this series. The different colors intend to individualize the different substituent groups of the molecule.

In 2023, Su and colleagues focused their efforts on the synthesis of a series of sixteen 1,3,5-triazine-quinoline hybrids and their evaluation as potential ChE inhibitors (**41**, Figure 18) [58]. Interestingly, depending on the substituent patterns, some molecules were either active against AChE or BuChE, while those displaying inhibitory activity against both enzymes present selectivity towards BuChE. For instance, compounds containing piperazine (IC_50_ 49.50 ± 0.81 μM and 63.62 ± 1.59 μM) or 4-(aminomethyl)piperidine (IC_50_ 21.43 ± 1.53 μM) as the linker between the quinoline and 1,3,5-triazine scaffolds only display activity against AChE. In turn, compounds containing 2-(aminomethyl)piperidine as the linker present inhibitory activity against both AChE and BuChE, with selectivity for BuChE. Regarding the other substitution on the 1,3,5-triazine scaffold, the pyrrolidine group appeared as the optimal substituent group against both enzymes, followed by diethylamine and dipropylamine (BuChE) or dipropylamine and dibutylamine (AChE).

**Figure 19:**
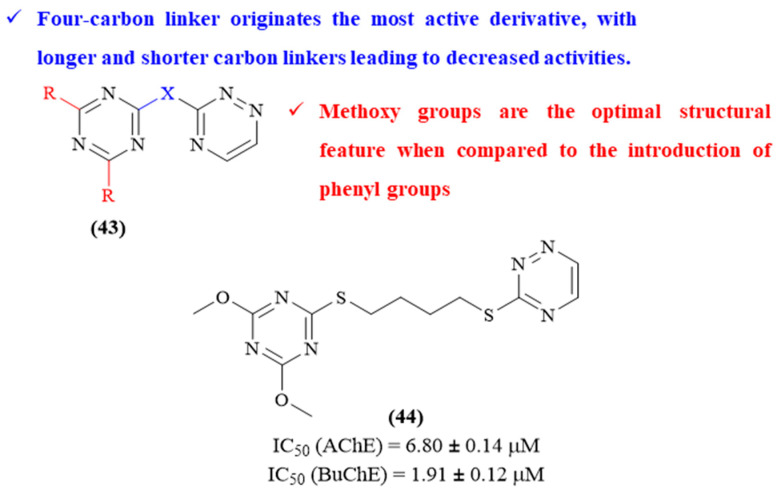
Structure–activity relationship of 1,3,5-triazine-1,2,4-triazine hybrids (**43**) against both AChE and BuChE, with emphasis on derivative **44** as the most active compound from this series. The different colors intend to individualize the different substituent groups of the molecule.

In the same year, another research group designed, synthesized and evaluated a novel series of 1,3,5-triazine-1,2,4-triazine hybrids towards their activity against both AChE and BuChE (**43**, Figure 19) [59]. From this series of hybrids, five molecules present considerable inhibition against AChE (IC_50_ values ranging from 6.80 ± 0.14 μM to 27.87 ± 0.26 μM), while only two molecules were able to significantly inhibit BuChE (IC_50_ values ranging from 1.91 ± 0.12 μM to 27.07 ± 0.24 μM). Structurally, it became clear that the linker length between the two scaffolds considerably affects the compounds’ AChE and BuChE-inhibitory activities, with the most active compound containing a four-carbon linker. Furthermore, the remaining substitutions on the 1,3,5-triazine ring also affect the compounds’ activity, with methoxy groups being the optimal structural feature compared to introducing phenyl groups. In this work, the authors also demonstrated that derivative **44**, the most active of the series against both enzymes, acts as a dual inhibitor by interacting with both CAS and PAS of AChE. In particular, the 1,3,5-triazine scaffold interacts via *π*–*π* stacking with Tyr337 of CAS, while the 1,2,4-triazine fragment interacts with Arg 296 via hydrogen bonding.

**Figure 20:**
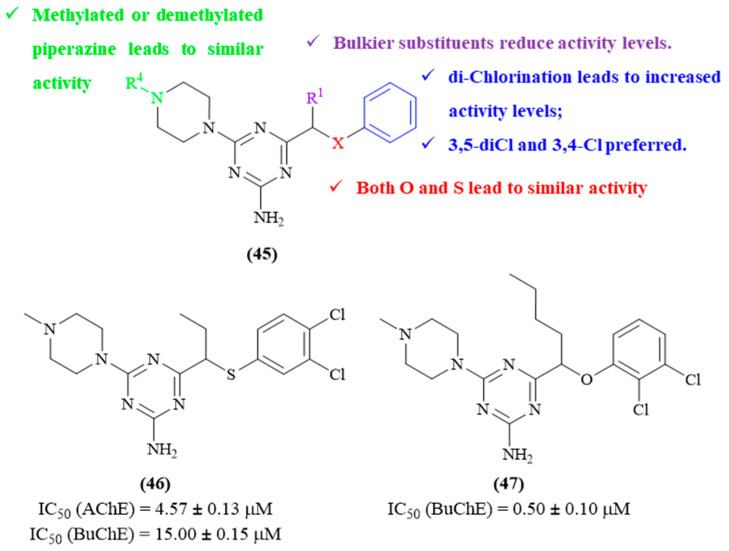
Structure–activity relationship of 1,3,5-triazine derivatives (**45**) against both AChE and BuChE, with emphasis on derivatives **46** and **47** as the most active compounds from this series. The different colors intend to individualize the different substituent groups of the molecule.

Still in 2023, Czarnota-Łydka and colleagues focused their efforts on the synthesis and biological evaluation of a series of thirty 1,3,5-triazine derivatives as potential multitarget compounds against AD (**45**, Figure 20) [60]. In addition to their inhibitory activity toward AChE and BuChE, these compounds were also evaluated as a potential ligand for 5-HT_6_ serotonin receptors (5-HT_6_R), a type of receptor that regulates the central cholinergic transmission through the modulation of GABA and glutamate levels on the central nervous system (CNS) [61]. However, in the sense of this paper, we will mostly focus on the effects of these derivatives on both AChE and BuChE. The results of this work demonstrate that six derivatives display considerable levels of activity against AChE (IC_50_ < 10 μM), with IC_50_ values ranging from 3.95 ± 0.16 μM to 9.52 ± 0.10 μM. In turn, only three were able to present significant values of BuChE-inhibitory activity, with IC_50_ values ranging from 0.50 ± 0.10 μM to 9.95 ± 0.20 μM. Interestingly, in both cases, these derivatives presented selectivity either for AChE or BuChE. From a structural perspective, four main structural features were evaluated, namely the influence of the heteroatom in the linker between the 1,3,5-triazine scaffold and the benzyl ring, the presence of substituents on the linker, the substitution pattern on the benzyl ring and the impact of the methyl group on the piperazine fragment. In particular, it was possible to verify that, regarding the heteroatom present in the linker, in general, both *O*- and *S*- originate derivatives with similar inhibitory activities. However, it was interesting to observe that, in some cases, this replacement led to a shift in the compound’s selectivity. Regarding the substituent groups on the linker, the introduction of bulkier substituents seems to considerably reduce the compounds’ inhibitory activity. With concerns to the substitution pattern of the benzyl ring, dichloro derivatives display higher levels of ChE-inhibitory activity than the monochloro or unsubstituted analogs. Furthermore, the position of these chloro atoms also affects the compounds’ inhibitory activity, with a 3,5-diCl and 3,4-Cl appearing to be preferred for this type of activity. Finally, the removal of the methyl group on the piperazine fragment seems to not have a significant impact on ChE inhibition, with both types of derivatives presenting similar levels of activity.

**Figure 21:**
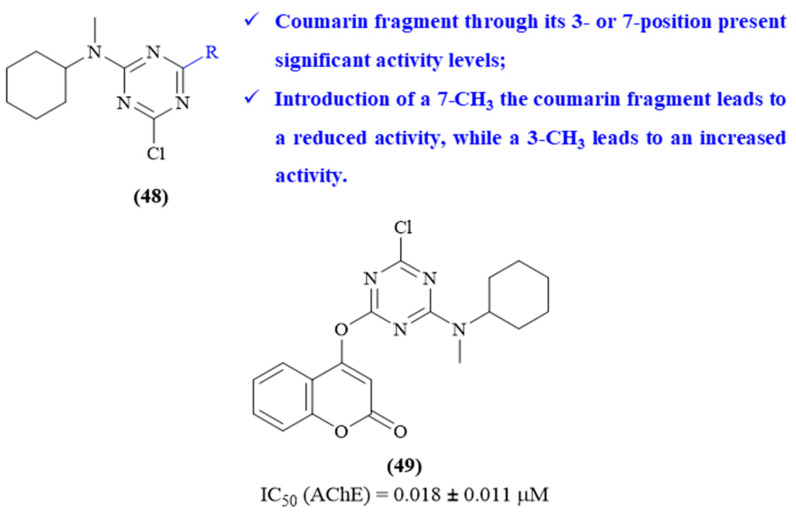
Structure–activity relationship of coumarin-1,3,5-triazine derivatives (**48**) against AChE, with emphasis on derivative **49** as the most active compound. The different colors intend to individualize the different substituent groups of the molecule.

In 2024, based on the potential of both coumarin and 1,3,5-triazine scaffolds, Zhang and colleagues designed and synthesized a series of six novel coumarin-1,3,5-triazine derivatives as AChE inhibitors (**48**, Figure 21) [62]. The results demonstrated that all the evaluated derivatives present considerable inhibitory activity against AChE (IC_50_ values ranging from 0.018 ± 0.011 μM to 12.48 ± 2.99 μM), with emphasis on derivative **49** (IC_50_ 0.018 ± 0.011 μM). From a structural perspective, it was possible to conclude that the position from which the coumarin fragment binds to the 1,3,5-triazine scaffold considerably affects the compounds’ inhibitory activity. For instance, the derivative containing the coumarin fragment bond to the 1,3,5-triazine scaffold through its 2-position presents no AChE-inhibitory activity whatsoever, while those containing the same bond through 3- or 7-position present significant activity levels (IC_50_ 0.018 ± 0.011 μM and 12.48 ± 2.99 μM, respectively). The introduction of a methyl group in the coumarin fragment promotes different effects depending on its position, with a 7-CH_3_ leading to a reduced activity (IC_50_ from 0.018 ± 0.011 μM to 0.82 ± 0.08 μM), while a 3-CH_3_ leads to an increased activity (IC_50_ from 12.48 ± 2.99 μM to 1.07 ± 0.20 μM). Finally, through some further studies, the authors also determined that derivative **49** displays a mixed-type inhibition and interacts with both PAS and CAS of AChE. More specifically, the 1,3,5-triazine scaffold interacts with TYR-124 and TYR-341 of PAS, through hydrogen and π-π stacking interactions, respectively, while the coumarin fragment interacts with GLY-82 and TYR-337 of CAS through hydrogen bond interactions, and with TYR-124 and TYR-341 of PAS through π-π stacking and a hydrogen bond interaction, respectively.

**Figure 22:**
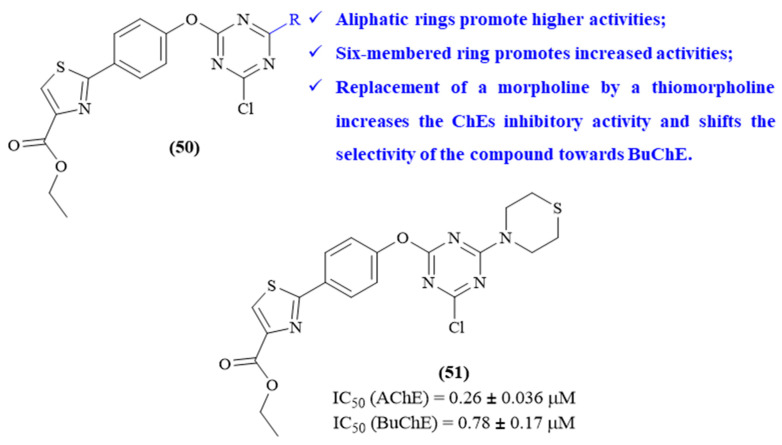
Structure–activity relationship of 2-phenylthiazole-1,3,5-triazine derivatives (**50**) against AChE and BuChE, with emphasis on derivative **51** as the most active compound. The different colors intend to individualize the different substituent groups of the molecule.

In the same year, the same research group synthesized a series of seven 2-phenylthiazole-1,3,5-triazine derivatives and evaluated them against both AChE and BuChE (**50**, Figure 22) [63]. In this work, five molecules display considerable levels of AChE-inhibitory activity (IC_50_ values ranging from 0.26 ± 0.036 μM to 35.06 ± 1.70 μM) and three molecules present significant BuChE-inhibitory activity levels (IC_50_ values ranging from 0.78 ± 0.17 μM to 11.65 ± 1.28 μM), with emphasis on derivative **51** as the most active compound against both enzymes. Interestingly, depending on the amino group introduced in the 1,3,5-triazine scaffold (R group), this type of molecule can present selectivity for AChE, selectivity for BuChE or no activity whatsoever. Structurally, verifying that the compound bearing aliphatic rings shows higher activity levels than the analog containing aromatic rings was possible. Furthermore, comparing five- or six-membered ring substituents, in general, the presence of a six-membered ring substituent promotes an increased level of AChE activity. Interestingly, the replacement of the oxygen by a sulfur atom in a morpholine not only increases the ChE-inhibitory activity but also shifts the selectivity of the compound towards BuChE, while the introduction of a di-methylated morpholine fragment leads to an increased AChE-inhibitory activity compared to an unsubstituted morpholine. Once again, the most active derivative was also studied through molecular docking to fully understand the compound–enzyme interactions, showing that this compound interacts with both active sites (PAS and CAS) of AChE. In this case, the phenylthiazole fragment interacts with PHE295, SER125 and TYR124, through hydrogen bonds, and with TYR337 and TYR341 through *π*-*π* stacking interaction. In turn, as already indicated throughout several other studies, the 1,3,5-triazine scaffold interacts with TRP86, through *π*-*π* stacking interactions.

**Figure 23:**
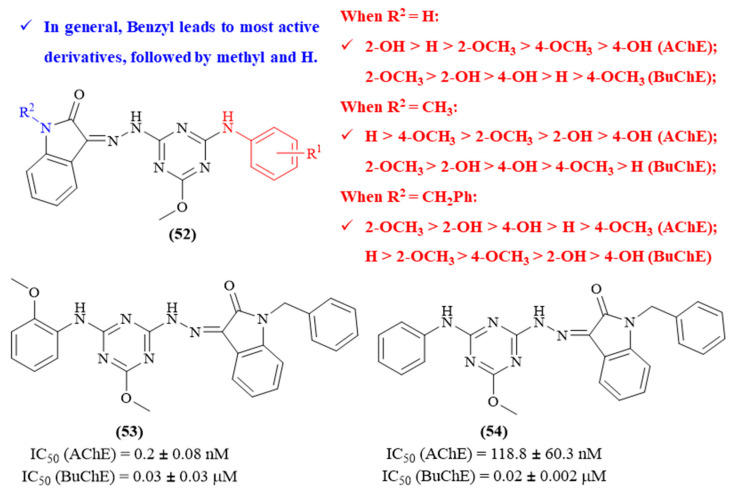
Structure–activity relationship of isatin-1,3,5-triazine hybrids (**52**) against AChE and BuChE, with emphasis on derivatives **53** and **54**. The different colors intend to individualize the different substituent groups of the molecule.

Still in 2024, Tamaddon-Abibigloo and colleagues focused their efforts on the synthesis of a series of fifteen new isatin-1,3,5-triazine hybrids and its evaluation as potential MTDLs against AD (**52**, Figure 23) [64]. The results demonstrated that this type of molecule displays an enormous potential against both AChE (IC_50_ values ranging from 0.2 ± 0.08 nM to 734.5 ± 44.4 nM) and BuChE (IC_50_ values ranging from 0.02 ± 0.002 μM to 1.92 ± 0.40 μM), with particular emphasis on the most active molecules (**53** and **54**). From a structural perspective, two main structural features were evaluated in order to deeply understand their effects on the compounds’ inhibitory activity against both ChEs, namely the substitution patterns of the aniline fragment and the isatin scaffold. Considering the AChE-inhibitory activity, when the isatin scaffold presented no substituent groups (R^2^ group), it was possible to verify that the presence of a 2-OH group on the aniline fragment leads to the highest level of activity, followed by the unsubstituted aniline, 2-OCH_3_, 4-OCH_3_ and 4-OH. When the R^2^ group is an *N*-methyl group, the most active derivative contains an unsubstituted aniline, followed by 4-OCH_3_, 2-OCH_3_, 2-OH and 4-OH. Finally, when the R^2^ group is an *N*-benzyl group, the presence of a 2-OCH_3_ leads to the most active molecule, followed by a 2-OH, 4-OH, unsubstituted aniline and 4-OCH_3_. When it comes to the compounds’ BuChE-inhibitory activity, even though still influenced by the substituent group present in the isatin (R^2^ group), the order of influence promoted by the aniline’s substitution pattern differs considerably from the observed for AChE. For instance, with an unsubstituted isatin, the presence of a 2-OCH_3_ leads to the most active molecule, followed by a 4-OH, 2-OH, unsubstituted aniline and 4-OCH_3_. When the R^2^ group is an *N*-methyl group, presence of a 2-OCH_3_ also originates the most active derivative, followed by a 2-OH, 4-OH, 4-OCH_3_ and unsubstituted aniline. Finally, when R^2^ group is an *N*-benzyl group, the most active molecule contains an unsubstituted aniline fragment, followed by 2-OCH_3_, 4-OCH_3,_ 2-OH and 4-OH. Further molecular docking studies also allowed for observing that most of these derivatives interact with TYR 124 and TRP 286 (PAS), TRP 86 (CAS) and TYR 341 (mid-gorge) of AChE. Interestingly, the compounds containing *N*-methyl or *N*-benzyl as R^2^ group present additional interactions with TYR 72 (PAS) and TYR 337 (CAS), with the *N*-benzylated derivatives displaying one more interaction with LEU289 (PAS), which might explain the generally higher activity demonstrated by these compounds.

## 3. Conclusions

With an increasingly aging population, dementia occupies a prominent place in our daily lives, with the search for a more effective treatment for AD becoming a focus for several research groups. In the search for better molecules against AD, the triazine scaffold emerges as one promising scaffold, particularly 1,3,5-triazines (*sym*-triazines), mainly due to the virtually unlimited possibilities of structural modifications that might be performed, allowing the obtention of derivatives that may interact with several molecular targets characteristic of this disease.

In this comprehensive review, we intended to highlight 1,3,5-triazines as potential agents for treating AD, focusing on their potential to act as MTDLs against this disease. Based on this, many SAR studies of 1,3,5-triazine derivatives were analyzed and compiled to better understand the optimal structural features to incorporate in the 1,3,5-triazine scaffold for each individual biological activity depicted. Considering the cholinergic hypothesis, the enzyme AChE remains the main molecular target, which reduces the presence of ACh in the system, with some relevance being highlighted for the role of BuChE in this system. On the other hand, in the amyloid hypothesis, two different approaches might be followed, the reduction in fibril formation by attacking them directly or the inhibition of the enzyme BACE-1 that consists of one of the cores of the disease. Additionally, the concept of MTDL is also explored throughout this review to provide enough information to develop a drug that might interact with not only one but several molecular mechanisms of this pathology simultaneously.

Throughout this review, numerous 1,3,5-triazine-based molecules have been depicted as displaying remarkable efficacy in targeting one or more hallmarks of AD, particularly by inhibiting AChE, BACE-1 or even modulating A*β*-aggregation. Based on the structural information compiled in the several SAR studies described in this review, it becomes possible to highlight some structural features that one can assume as optimal for each of the desired biological activities. For instance, regarding the inhibition of AChE, the introduction of acetylcholine-like fragments or anilines containing electron-withdrawing groups, like trifluoromethyl groups and fluorine atom, often led to a significant activity increase. Considering the inhibition of BACE-1, the introduction of some specific amino groups, like pyrrolidine or piperidine, seems to be a structural feature to be considered for the inhibition of BACE-1 and, in turn, the incorporation of steric bulk substituents. Finally, it became clear that the hybridization of the 1,3,5-triazine scaffold with other pharmacophores, such as triazolopyrimidine, benzothiazole, quinoline, isatin, phenylacetamide, amongst others, emerged as a promising approach for the development of MTDL agents against different molecular targets from AD.

To conclude, especially in the past two decades, the 1,3,5-triazine scaffold has been intensively studied to develop novel treatments, and some successful preliminary results have already been demonstrated. Based on this, the potential use of 1,3,5-triazines for developing novel MTDL against AD becomes undeniable, with this scaffold presenting a versatile and promising foundation for this purpose. However, there is still a long way to go to design and develop an efficient treatment for AD, with most of the molecules depicted throughout this manuscript still lacking data concerning in vivo and clinical studies, hampering the full understanding of this family’s potential against this disease. Furthermore, despite all of the studies described in this manuscript highlighting the potential of 1,3,5-triazines against AD, little to no information is given regarding their BBB permeability, one of the main challenges for the development of drugs against neurodegenerative disease. Thus, even though there is already a considerable number of studies performed on 1,3,5-triazines, further developments and the synthesis of a bigger library of 1,3,5-triazines are still necessary to allow a complete overview of the use of these molecules for more comprehensive and effective therapeutic options for this devastating neurodegenerative disorder.
